# A new approach for clinical translation of infrared spectroscopy: exploitation of the signature of glioblastoma for general brain tumor recognition

**DOI:** 10.1007/s11060-022-04204-3

**Published:** 2022-12-12

**Authors:** Gerald Steiner, Roberta Galli, Grit Preusse, Susanne Michen, Matthias Meinhardt, Achim Temme, Stephan B. Sobottka, Tareq A. Juratli, Edmund Koch, Gabriele Schackert, Matthias Kirsch, Ortrud Uckermann

**Affiliations:** 1 Clinical Sensoring and Monitoring, Department of Anesthesiology and Intensive Care Medicine, Faculty of Medicine, TU Dresden, Dresden, Germany; 2 Medical Physics and Biomedical Engineering, Faculty of Medicine, TU Dresden, Dresden, Germany; 3 Department of Pathology (Neuropathology), University Hospital Carl Gustav Carus, TU Dresden, Dresden, Germany; 4 Department of Neurosurgery, University Hospital Carl Gustav Carus, TU, Dresden, Germany; 5 National Center for Tumor Diseases (NCT), Partner Site Dresden, German Cancer Research Center (DKFZ), Heidelberg, Germany; 6 German Cancer Consortium (DKTK), Partner Site Dresden, and German Cancer Research Center (DKFZ), Heidelberg, Germany; 7 Asklepios Kliniken Schildautal Seesen, Seesen, Germany; 8 Division of Medical Biology, Department of Psychiatry and Psychotherapy, Faculty of Medicine and University Hospital Carl Gustav Carus, TU Dresden, Fetscherstr. 74, 01307 Dresden, Germany

**Keywords:** Infrared spectroscopy, Brain tumor, Delineation, Intraoperative

## Abstract

**Purpose:**

Infrared (IR) spectroscopy has the potential for tumor delineation in neurosurgery. Previous research showed that IR spectra of brain tumors are generally characterized by reduced lipid-related and increased protein-related bands. Therefore, we propose the exploitation of these common spectral changes for brain tumor recognition.

**Methods:**

Attenuated total reflection IR spectroscopy was performed on fresh specimens of 790 patients within minutes after resection. Using principal component analysis and linear discriminant analysis, a classification model was developed on a subset of glioblastoma (n = 135) and non-neoplastic brain (n = 27) specimens, and then applied to classify the IR spectra of several types of brain tumors.

**Results:**

The model correctly classified 82% (517/628) of specimens as “tumor” or “non-tumor”, respectively. While the sensitivity was limited for infiltrative glioma, this approach recognized GBM (86%), other types of primary brain tumors (92%) and brain metastases (92%) with high accuracy and all non-tumor samples were correctly identified.

**Conclusion:**

The concept of differentiation of brain tumors from non-tumor brain based on a common spectroscopic tumor signature will accelerate clinical translation of infrared spectroscopy and related technologies. The surgeon could use a single instrument to detect a variety of brain tumor types intraoperatively in future clinical settings. Our data suggests that this would be associated with some risk of missing infiltrative regions or tumors, but not with the risk of removing non-tumor brain.

**Supplementary Information:**

The online version contains supplementary material available at 10.1007/s11060-022-04204-3.

## Introduction

Modern neurosurgery focuses on safe resection and sparing of healthy tissue to maintain quality of life. Moreover, the extent of resection is linked to patient’s survival for different brain tumors [1–4]. Hence, brain tumor resection is often a balancing act between removing suspicious tissue and preserving the functional relevant brain substance. Numerous approaches such as mapping with somatosensory-evoked potentials, magnetic resonance imaging, ultrasound and 5-ALA fluorescence microscopy are used intraoperatively to improve tumor delineation and to optimize the extent of resection [5–7]. However, some of these tools are limited to certain tumor types and the precise identification of tumor borders remains challenging. Therefore, other objective techniques based on molecular characteristics need to be integrated into the surgical procedures to achieve the goal of maximal tumor resection and simultaneously enable rapid and reliable diagnosis in situ.

Many studies demonstrated that methods of vibrational spectroscopy, like infrared (IR) and Raman spectroscopy, have the potential to provide relevant diagnostic information on tumors [8] including cerebral neoplasms [9, 10]. They are well suited for clinical use because being pure optical damage-free techniques that do not require tissue processing nor staining. Basic research on experimental brain tumor models [11] as well as on human brain tumors [9, 12, 13] identified reduced lipid-related bands and increased protein-related bands in IR spectra of all investigated brain tumors types (including glioma, meningioma and metastases) compared to spectra of non-tumor brain parenchyma. The basic requirements for clinical translation of IR spectroscopy with respect to the processing of spectral data sets were defined [14] and concepts for resection guidance have been developed [15]. The potential of vibrational spectroscopy has already been demonstrated also for in vivo applications and confirmed as real-time diagnostic method [16, 17]. Raman spectroscopy can be used to detect tumor infiltrations beyond areas of 5-ALA fluorescence [18] highlighting the great potential of vibrational spectroscopy for intraoperative delineation of brain tumors and resection guidance. However, a real clinical application of IR or Raman spectroscopy is still lacking despite the very promising results [19, 20]. One reason might be the limited availability of reference data, especially for less frequent tumors, and the lack of a clinically manageable approach to overcome this issue in the future.

We propose to exploit the similarity of spectral changes in different types of brain tumors for clinical translation and tested our hypothesis using IR spectroscopy on a large set of fresh brain tumor specimens. Given the common spectral properties of brain tumors, i. e. reduced lipid-related band and increased protein-related bands, we hypothesize that the spectral signature can be identified from data sets of frequent tumors and then exploited for universal tumor detection in clinical applications. In this study, glioblastoma (GBM) was selected to provide reference data because (i) it is the most frequent primary brain tumor in adults with high availability of specimen material and (ii) it encompasses a variety of histologic features. First, a strategy for discrimination of non-tumor brain and GBM was developed taking into account inter-patient variability. In a second step, this algorithm was tested for classification of other brain tumors types.

We report the results of the first large study of 790 patients on intraoperative delineation between brain tumors and normal tissue. Infrared spectra taken from small amounts of freshly resected tissue indicate the presence of normal tissue or brain tumor within approximately 1 minute. We describe an approach for clinical spectra analysis and a comprehensive benchmarking framework with broader scale and scope than previously reported.

## Materials and methods

### Study design

Study objectives were first, the assessment of IR spectral changes of brain tumors of fresh samples and second the development of a toolbox for interpretation of spectral signatures of unknown surgical specimens. The study was approved by the ethics committee at TU Dresden (EK 323,122,008). The study spanned 7.5 years (08/2011–02/2019) and inclusion criteria were: (i) female or male adults; (ii) routine brain tumor extirpation or surgeries for the treatment of pharmacoresistant epilepsy at the Department of Neurosurgery, University Hospital Carl Gustav Carus at the TU Dresden, Germany; (iii) written consent of the patient; (iv) availability of excess removed tissue material that was not needed for diagnostics or other clinical purposes.

## Infrared spectroscopy of fresh samples

Tissue specimens were obtained directly from the neurosurgeon. The IR spectroscopic measurements were performed by a study nurse or medical technical assistant who was shortly instructed about use of the spectroscopic device.

Attenuated total reflection (ATR) Fourier-transform IR spectra of fresh, unprocessed tissue were acquired using an ALPHA-P ATR spectrometer (Bruker Optics GmbH) equipped with diamond crystal as described elsewhere [15]. A spectral resolution of 4 cm^−1^ and averaging of 128 spectra were set, resulting in an acquisition time of ~ 1 min. The measured sample was handed over to routine diagnostic histopathology after measurement.

## Data analysis

Spectra were reduced to the range of 1000–1480 cm^−1^. The spectral range of the amide I and amide II bands (1480–1700cm^−1^) was not taken into account, since strong absorptions occur here due to the deformation vibration mode of free water. Even slight variations in the water content of the tissue, together with the penetration depth of the evanescent field, lead to strong changes in the spectrum. Furthermore, previous studies have shown that relevant signals for the differentiation of brain tissue and brain tumors do occur outside the region of the amide I and amide II bands [21–23]. Spectral artifacts related to absorption bands of water vapor were corrected using the function *atmospheric compensation* of OPUS software package (Bruker Corp. MA, USA) and a quadratic baseline procedure was performed as described elsewhere [15]. Further analysis of spectra was performed with MATLAB R2020a (The MathWorks Inc., Natick, MA, USA). The spectra were vector normalized. Statistical analysis of band intensities was performed using Mann–Whitney test in Graph Pad Prism 8 (GraphPad Software, San Diego, CA, USA).

## PCA and selection of principal components on the training set

Principal component analysis (PCA) was used to reduce data dimensionality. PCA was performed exclusively on spectra of the training set (non-tumor and GBM). The first 14 PCs were selected based on performance analysis (see supporting material, Supporting Figure S1, S2).

## Calculation of PC scores of test set based on given PC loadings of the training set

Besides providing loadings and scores, the MATLAB function “pca” provides the estimated mean of each variable in x that was employed to transfer the PCA obtained from the training data set to test data. PC scores of test data were obtained by subtracting this value from the spectra and multiplying by the coefficient for PCs 1–14, respectively.

## Classification model

The classification model was developed on the training data set (only GBM and non-tumor) and then applied to the independent test data. Linear discriminant analysis (MATLAB function “classify”) was employed to classify PC scores and provided the probability of class assignment for each spectrum. For non-tumor samples, multiple measurements were performed, each spectrum was classified, and the mean of probabilities was calculated providing the classification result for the sample.

## Results

751 patients undergoing brain tumor extirpation (median age 61, 297 females and 415 males) and 39 patients with surgeries for the treatment of pharmacoresistant epilepsy (median age 39, 17 females and 22 males) were included into the study. Fresh tissue specimens were examined immediately after resection. The majority of samples was analyzed within a few minutes after resection (median 6 min, 25% percentile: 3 min, 75% percentile: 10 min). The cohort comprised patients diagnosed with glioma WHO I-IV, other types of primary brain tumors and brain metastases of peripheral cancers and is summarized in Table 1. Notably, only one spectrum was acquired for each tumor sample to keep the workflow simple, fast and manageable for the OR staff.

**Table 1:** Overview of samples and their assignment to test and training set. The patient was diagnosed by routine histopathological examination according to the WHO classification of brain tumors that was valid at the time of resection. For non-tumor tissue, multiple measurements were performed on one sample and the number of acquired spectra is given in parenthesis. From each tumor biopsy, one spectrum was acquired

Group	n (Patients)		Patient’s diagnosis
Non-tumor	27	Training (44 spectra)	Ammon’s horn sclerosis, dysplasia, normal tissue
	12	Test (47spectra)	
GBM WHO IV			Glioblastoma
Primary disease	135	Training	
	134	Test	
Recurrent	115	Test	Recurrent glioblastoma or glioblastoma with history of lower grade glioma
Glioma WHO III	83	Test	Anaplastic astrocytoma or oligodendroglioma
Glioma WHO II	16	Test	Diffuse astrocytoma or oligodendroglioma
Glioma WHO I	6	Test	Pilocytic astrocytoma
Other tumors	87	Test	Choroid plexus tumors, embryonal tumors, endocrine tumors, ependymal tumors, germ cell tumor, lymphomas, mesenchymal non-meningiothelial tumors, neuronal and mixed neuronal-glial tumors, primary melanocytic lesion, tumors of sellar region
Metastases	175	Test	Brain metastases of colon carcinoma, lung carcinoma, mamma carcinoma, Malignant melanoma, renal carcinoma, other primum (adnexa, bladder, ovary, pancreas, prostate, rectum, sarcoma, thyroid, stomach) or unknown
TOTAL	**790**		

## Classification of glioblastoma and non-tumor brain tissue

**Fig. 1:**
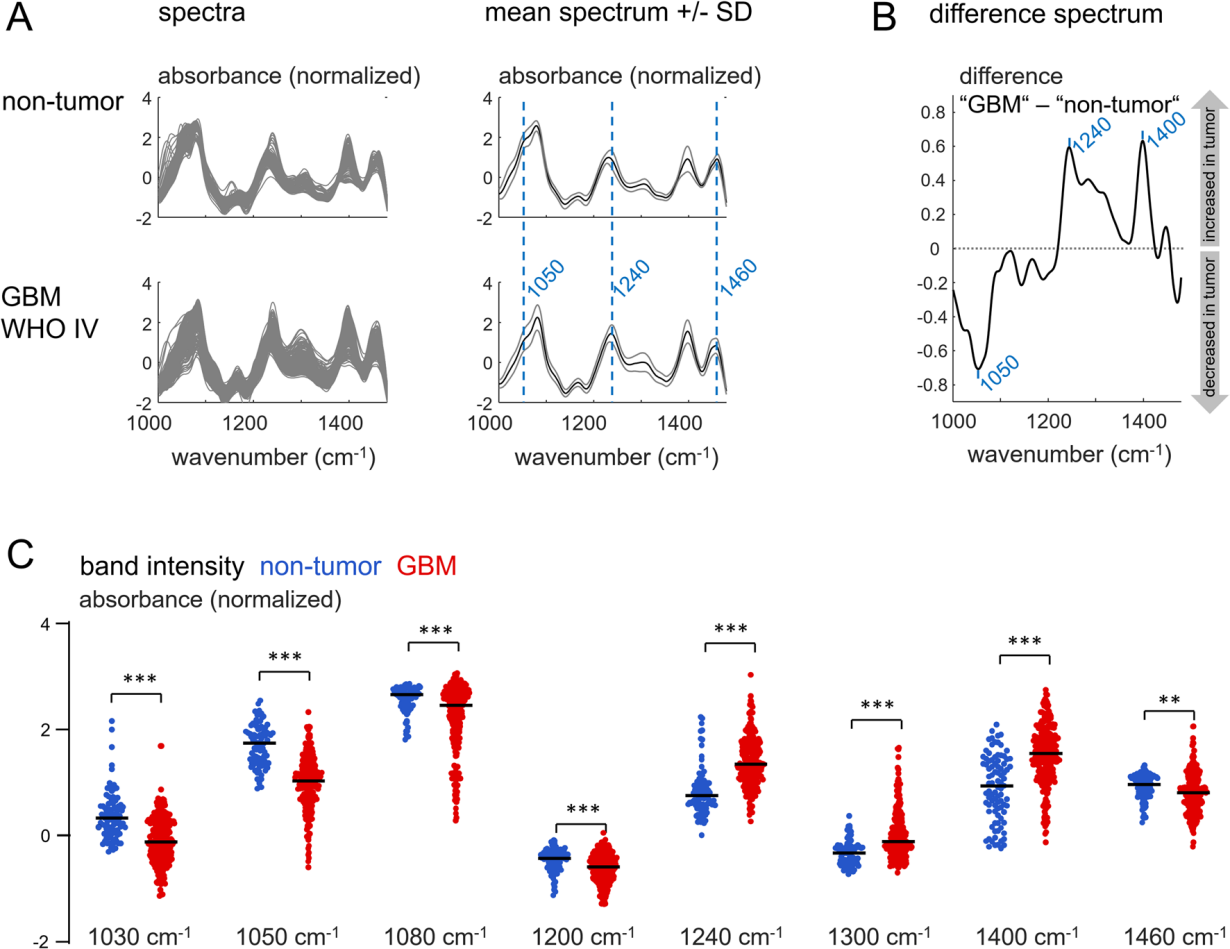
Spectral characteristics of human GBM in comparison to non-tumor brain: **A** Preprocessed spectra and mean spectra **B** mean difference of GBM and non-tumor spectra **C** Intensities of selected IR bands for non-tumor and GBM. Points represent spectroscopic measurements (non-tumor: multiple measurements on 39 patient’s biopsies; GBM: one measurement was performed on each biopsy), black line indicates the median. Significant difference ***P < 0.001 (Mann–Whitney test)

Specimens of 269 GBM patients (median age: 68, 113 females and 156 males) were analyzed and compared to the spectra of non-tumor brain tissue. The raw ATR infrared spectrum of fresh tissue is dominated by large bands of water (Supporting Figure S3). These were excluded from the analysis, leaving only the spectral region containing the bands associated with tissue components that are possible markers for tumors [15, 24, 25]. Figure 1A shows the preprocessed spectra (left) and calculated mean spectra (right) for non-tumor samples and GBM tissue. The spectra of non-tumor and tumor tissue show likewise the main bands typical for nervous tissue, which are assigned to carbohydrates, nucleic acids, (phospho-)lipids and proteins as reported in Supporting Table S1. Differences between non-tumor and GBM tissue became apparent upon comparison of the mean spectra. The bands around 1050 and 1460 cm^−1^ display different shapes and the maximum of the band at 1240 cm^−1^ was shifted towards higher wavenumbers in tumors (blue dashed lines in Fig. 1A). The difference of mean spectra of GBM and non-tumor tissue indicates that the band around 1050 cm^−1^ is reduced in GBM, while the band intensities at 1240 and 1400 cm^−1^ are increased (Fig. 1B).

The intensities of selected IR bands are shown in Fig. 1C for all GBM and non-tumor spectra (line indicates median). The bands related to (phospho- /glyco-) lipids [26–28] around 1030, 1050, 1080, 1200 and 1460 cm^−1^exhibit significantly smaller intensities in GBM than in non-tumor tissue. The bands at 1240, 1300 and 1400 cm^−1^ are associated with proteins [28, 29] and display significantly increased intensities in the GBM samples. These findings are in accordance with previous studies indicating that GBM display reduced lipids and increased protein profiles [9, 12, 13].

For clinical exploitation, the IR spectrum of an unknown biopsy has to be assigned to the correct class based on information obtained from reference data. An established approach for spectra classification involves (i) the dimensionality reduction of the data, which exploits only spectral features carrying the desired information, (ii) the development of a classification model using training data and (iii) the application of this classification model on new data sets (test set) [30].

The patients’ diagnoses according to the WHO classification of brain tumors that was valid at the time of resection were used as ground truth for labelling of spectra. Spectra from a patient’s specimen were assigned to either the training or the test set. The training set was built with spectra acquired from biopsies of GBM brain tumors (n = 135 spectra, 135 patients) and non-tumor brain tissue (n = 44 spectra, 27 patients). In the test set, spectra of different primary and secondary brain tumors (n = 616 spectra, 616 patients) and non-tumor brain tissue (n = 47 spectra, 12 patients) were analyzed (Table 1).

**Fig. 2:**
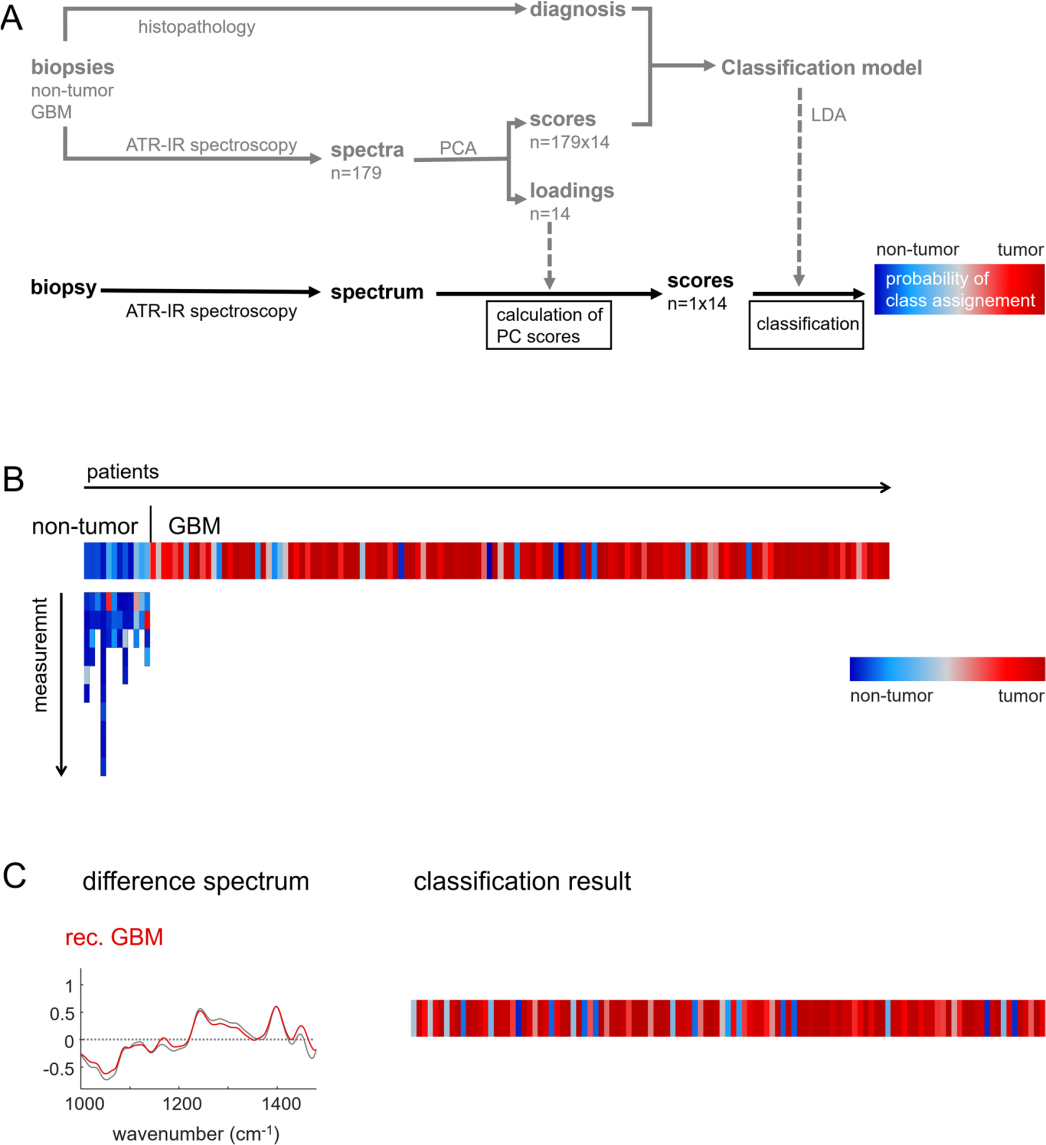
Classification of GBM versus non-tumor specimens **A** Developed data analysis strategy that is compatible with future clinical exploitation (PCA: principal component analysis, LDA: Linear discriminant analysis) **B** Classification result for non-tumor samples and GBM (primary disease, samples independent of the training set). Multiple measurements on the same sample were performed for non-tumor tissue. **C** Difference spectra to non-tumor of recurrent GBM (red) and primary disease GBM (gray) **D** Classification result for specimens of recurrent GBM. The probability of class assignment is shown for each patient in a color code

The band intensities shown in Fig. 1C reveal the large interpatient variability. It is not possible to assign a specific spectrum based on a selected absorbance to a either non-tumor or GBM because of the presence of large overlaps. This implies that the diagnostic exploitation of the technology requires consideration of the entire spectral range. Therefore, we decided to perform principal component analysis (PCA), as an approach for preserving spectral information while reducing data dimensionality. Half of the spectra obtained on non-tumor tissue and GBM samples (i. e. training set, see Table 1) were used as input for PCA. The scores of the PC 1–14 and the given patient’s diagnosis were then used to build a classification model based on linear discriminant analysis, which provides the probability for being non-tumor or tumor for a specimen (Fig. 2A, gray flowchart, see Supporting Figure S4 for reclassification of the training set).

Spectra of the test set are representative for new, unknown samples in future clinical applications and were neither included in PCA nor used for the development of classifier (Fig. 2A, black flowchart). The classification model was tested for 12 non-tumor and 134 GBM WHO IV specimens of the test set (Fig. 2B). All 12 non-tumor samples and 118/134 (88%) of GBM samples were correctly recognized. Additionally, the strategy was validated on a group of recurrent GBM (115 patients, median age: 61, 49 females and 66 males). Most of those patients (104/115) underwent standard therapy for treatment of GBM that constitutes of tumor resection followed by radiation and chemotherapy with temozolomide. The remaining 11 patients either had a diagnostic biopsy followed by radio-chemotherapy (n = 2) or without further therapy (n = 1), or tumor resection followed by radiotherapy only (n = 3), by chemotherapy only (n = 1) or without adjuvant therapies (n = 4). The difference spectra of recurrent GBM and non-tumor brain confirmed very similar changes like in primary disease GBM (Fig. 2C). The algorithm correctly classified 95/115 (83%) of recurrent glioma as tumor (Fig. 2C). No evidence of a correlation between prior anti-cancer therapy or its absence and classification outcome was found. These results demonstrate that PCA-based analysis and the exploitation of IR spectra for fast tumor delineation can be performed in a clinical context with high reliability despite the large interpatient variability of GBM and previous therapy, respectively.

## Different types of brain tumors

Analysis of spectral changes compared to non-tumor tissue and testing of the classification model developed on GBM were then performed for several types of brain tumors. This included glioma WHO III (83 patients, median age: 41, 33 females and 50 males), glioma WHO II (16 patients, median age 39, 9 females and 7 males), pilocytic astrocytoma (6 patients, median age 19, 2 females and 4 males), other primary brain tumors (87 patients, median age 50, 35 females and 52 males) and brain metastases (175 patients, median age 63, 73 females and 102 males). Both, patient age and gender distribution are consistent with the known epidemiology of the corresponding type of primary brain tumors [31].

**Fig. 3:**
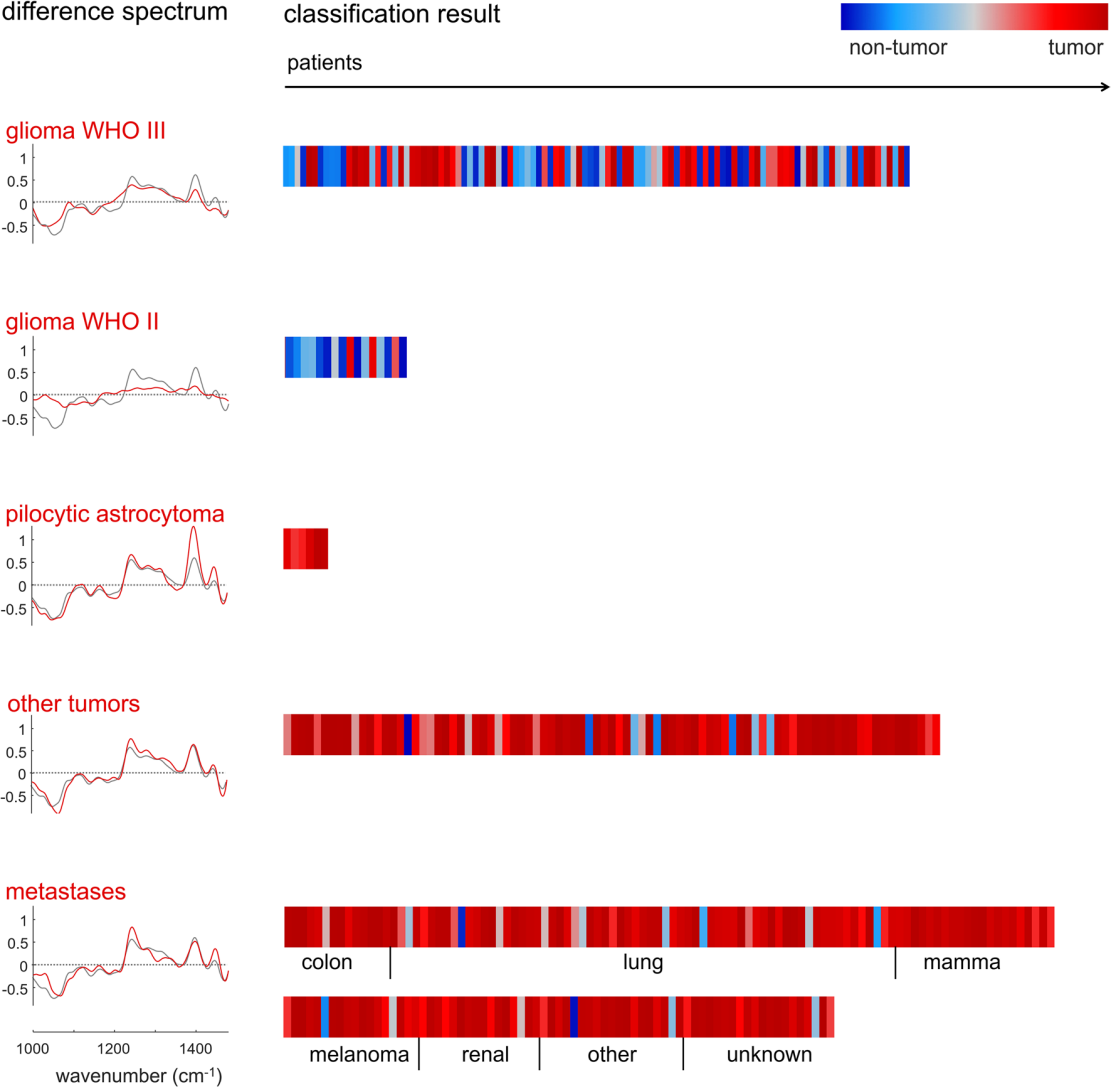
Difference spectra to non-tumor brain tissue and classification results for different types of brain tumors. Difference spectra of the brain tumor type (red) are shown in comparison to the difference spectrum of GMB and non-tumor tissue (gray), respectively. The classification result shows the probability of class assignment for each patient in a color code. The primum of brain metastases is indicated

Figure 3 (left) shows the difference spectra of the respective group of brain tumor and non-tumor tissue (red) in comparison to the difference spectrum of GBM and non-tumor brain (gray). Spectral differences to non-tumor brain tissue were less pronounced in glioma WHO III and almost absent for glioma WHO II. However, the difference spectra of pilocytic astrocytoma, other primary brain tumors and brain metastases are similar to the difference spectra of GBM.

Figure 3 (right) shows the performance of the algorithm for recognition of a certain type of tumor. Only a few glioma WHO II (3 of 16) and half of the glioma WHO III samples (40/83) were correctly classified. However, this approach was well suited for pilocytic astrocytoma (all 6 samples), other types of primary brain tumors (80/87 samples, correct rate 92%) and for metastases of several peripheral cancers (163/175 samples, correct rate 93%). This supports our hypothesis that brain tumors with similar spectral changes can indeed be classified based on algorithms established on reference data of different tumor types. In line with this finding, the analysis of misclassified specimens revealed, that those display indeed spectral characteristics similar to non-tumor brain tissue (supporting material, supporting Figures S5,6).

## Discussion

In this study, fresh tumor biopsies were investigated by ATR IR spectroscopy to bridge the gap between basic research and clinical exploitation by development of strategies for intraoperative brain tumor delineation in neurosurgery. Our data shows that IR spectra of fresh brain tumor biopsies can be obtained routinely during surgery. They were acquired within a few minutes after resection using a small sturdy device that was operated by a trained study nurse. We obtained useful spectral quality and consistent data over several years and show that contributions of water in the IR spectrum do not hinder exploitation for brain tumor delineation. This proves the reliability of the approach, which is of utmost importance for clinical implementation.

As we were able to analyze samples of almost 800 patients, the large variability of the spectral data sets became visible, which can be explained by the well-known interpatient variability. This has important consequences for the development of strategies for clinical evaluation, as simple diagnostic strategies based on the definition of threshold values or the analysis of band intensities cannot be applied. Merging the two concepts of (i) considering the full spectral range exploiting PCA for dimensionality reduction and (ii) using only GBM tumor signature as reference, we developed an innovative strategy that complies with future clinical exploitation.

In accordance to previous research [9, 12, 13], we confirmed for fresh specimens that the different brain tumor types investigated (including primary and secondary brain tumors) share similar changes in their IR spectroscopic signature. Although the exact band positions and magnitude of changes varied, we identified a clearly recognizable pattern: IR bands related to lipids were decreased in primary and secondary brain tumors and bands related to proteins were increased. In glioma WHO II and WHO III, average spectral changes were smaller compared to non-tumor tissue. This might reflect the smaller degree of remodeling of tissue architecture in these tumors, which is consistent with their histopathology.

Considering all tumor specimens of the training set (including glioma WHO II and III) 517/628 (82%) of specimens were correctly classified. Our results lie in similar range with existing studies, even if comparison of classification performance is not straightforward because of differences in instrumentation, sample preparation or experimental groups. Classification using genetic algorithm with LDA on ATR-IR spectra of frozen sections resulted in an accuracy of 79.2% for glioma [32]. Using IR imaging on frozen tissue sections, tissue components were predicted with an accuracy of 85.2% for brain metastases [33] and human glioma grade III and IV were identified with correct rate of 81.7% [10]. For classification of glioma WHO I-III, glioblastoma and control tissue, a success rate of 89% of spectra on 22 samples was obtained [22]. Single IR spectra obtained from tissue cryosections were correctly assigned to normal brain tissue with 100% accuracy and to malignant glioma with 93% accuracy [26].

The detection of brain tumors with less pronounced spectral changes compared to non-tumor brain tissue remains a limitation of our approach. The classifier developed on GBM and non-tumor tissue was not effective for recognition of these tumors. However, it has to be considered that the number of non-tumor brain specimens included in the presented study is small and was obtained from patients undergoing epilepsy surgery. Therefore, it might have provided only an approximation of the spectral signature of the parenchyma around the tumor. The application of vibrational spectroscopy in situ would largely improve the availability of spectral data of non-tumor brain tissue and might allow defining more accurately spectral signature of peritumor tissue. Moreover, non-tumor and tumor regions of the same patient could be analyzed in situ in a personalized manner. It remains an open question whether this would allow the refinement of the present approach and lead to improved classification accuracy or whether it will be necessary to extract a specific signature of glioma WHO III and II.

Despite the limitations and open questions described above, we suggest the exploitation of a general spectral brain tumors signature for translational applications of IR spectroscopy. This general spectral brain tumor signature could be extracted from data sets of frequent tumors and then used for universal tumor classification. Importantly, this approach might likewise be important for Raman spectroscopy, which also probes biochemical tissue composition and likewise revealed similar spectral changes for different types of brain tumors including GBM, low grade glioma and brain metastases [34]. If validated, this concept could significantly advance the routine clinical use of IR and Raman spectroscopy and make them available also for delineation of rare tumors.

In future research, we envision the spectral analysis of brain tissue in situ during surgery. From a technical point of view, this can be easily implemented in the clinical workflow, as acquisition times are short (seconds). ATR IR spectroscopy [35]and Raman spectroscopy [36] can be performed using fiber-based systems that were already demonstrated to provide high quality spectra of human brain tumors. Moreover, we have already shown that prognostic relevant molecular markers like *IDH1* mutation and tumor stem cells can be addressed by both techniques, with large potential for intraoperative tumor characterization [17, 25, 37]. Previous work using IR and Raman spectroscopy suggest that different types of brain tumors are characterized by distinct spectroscopic signatures [26, 34, 38]. By increasing the number of patients, the large variability found in this study could be compensated and machine learning could exploit the tumor-specific signature for diagnostics. Here, we provide a path to the clinic that seems manageable and gives stable results. Our results lay the groundwork for future clinical studies that will investigate whether intraoperative clinical spectroscopy actually translates into patient benefit, i.e., longer progression free and overall survival or improved quality of life.

## Supplementary Information

Below is the link to the electronic supplementary material.

Supplementary file1 (PDF 735 kb)

## Data Availability

The datasets generated during the current study are available from the corresponding author on reasonable request.
