# Supplementary material for: A new approach for clinical translation of infrared spectroscopy: exploitation of the signature of glioblastoma for general brain tumor recognition
Source: J Neurooncol. 2022 Dec 12;161(1):57–66. doi: 10.1007/s11060-022-04204-3 (PMC9886632; doi:10.1007/s11060-022-04204-3)
Supplement: Supplementary file 1 — Supplementary file1 (PDF 735 kb) [file 11060_2022_4204_MOESM1_ESM.pdf]

SUPPORTING MATERIAL AND FIGURES

Journal of Neuro-Oncology

Title:

A new approach for clinical translation of infrared spectroscopy: exploitation of the signature of glioblastoma for general brain tumor recognition

Authors:

Gerald Steiner[1], Roberta Galli[2], Grit Preusse[1], Susanne Michen[4], Matthias Meinhardt[3], Achim Temme[4,5,6,], Stephan B. Sobottka[4], Tareq A. Juratli[4], Edmund Koch[1], Gabriele Schackert[4,5,6,], Matthias Kirsch[7], Ortrud Uckermann[4,8]

Affiliations:

[1]Clinical Sensoring and Monitoring, Department of Anesthesiology and Intensive Care Medicine, Carl Gustav Carus Faculty of Medicine, TU Dresden

[2]Medical Physics and Biomedical Engineering, Carl Gustav Carus Faculty of Medicine, TU Dresden

[3]Department of Pathology (Neuropathology), University Hospital Carl Gustav Carus at TU Dresden

[4]Department of Neurosurgery, University Hospital Carl Gustav Carus at TU Dresden

[5]National Center for Tumor Diseases (NCT), Partner Site Dresden, German Cancer Research Center (DKFZ), Heidelberg, Germany

[6]German Cancer Consortium (DKTK), Partner Site Dresden, and German Cancer Research Center (DKFZ), Heidelberg, Germany

[7]Asklepios Kliniken Schildautal Seesen, Seesen, Germany

[8]Division of Medical Biology, Department of Psychiatry and Psychotherapy, Medical Faculty and University Hospital Carl Gustav Carus, TU Dresden, Germany

Corresponding author:

Ortrud Uckermann, Medical Biology, Medical Faculty, TU Dresden, Fetscherstr. 74, 01307 Dresden, Germany, telephone +49 531 458 3114, fax: +49 531 458 4304 ortrud.uckermann@uniklinikum-dresden.de,

Performance analysis

PC scores were used as input for linear discriminant analysis using a leave-one-out strategy. The area under the ROC curve (AUC) was calculated to evaluate the classification model's performance to find the minimal number of PCs that sufficiently describe the spectral dataset for discrimination of GBM and non-tumor tissue (Supporting Figure S1). We found a maximal AUC of 0.90 using the scores of the first 14 PCs (Supporting Figure S2). They account for 99.3% of the variance in the dataset. Using this procedure, each spectrum is now represented by 14 values instead of 339 data points.

PCA, classification and calculation of receiver operating characteristic (ROC) curve were performed using standard MATLAB functions "pca", "classify" and "roc" respectively.

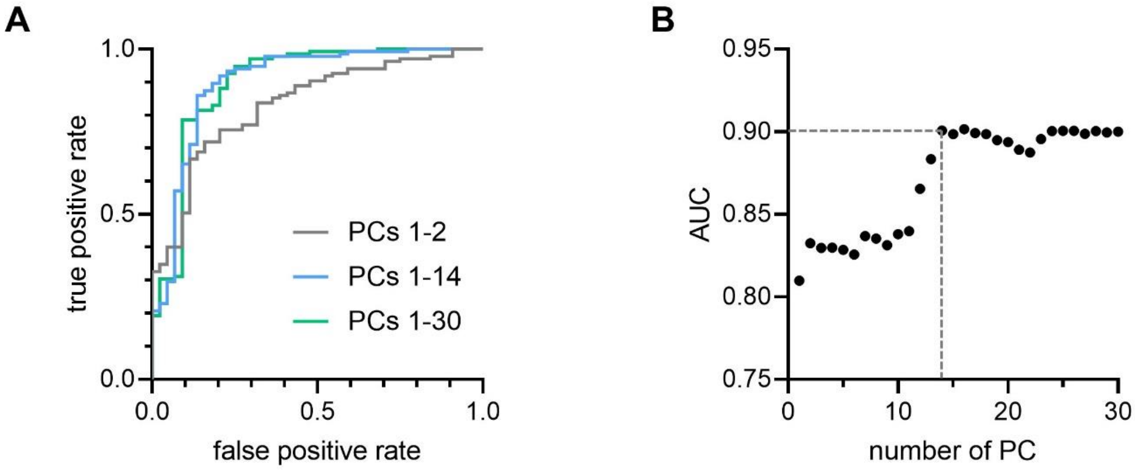

Figure S1: Evaluation of the performance of the classification tumor versus non-tumor of the training set. A leave-one-out approach using the scores of principal components (PCs) was applied. **A:** Receiver operating characteristic (ROC) curve for PCs 1-2, 1-16 and 1-30. **B:** Area under the ROC curve (AUC) using different number of principal components for classification.

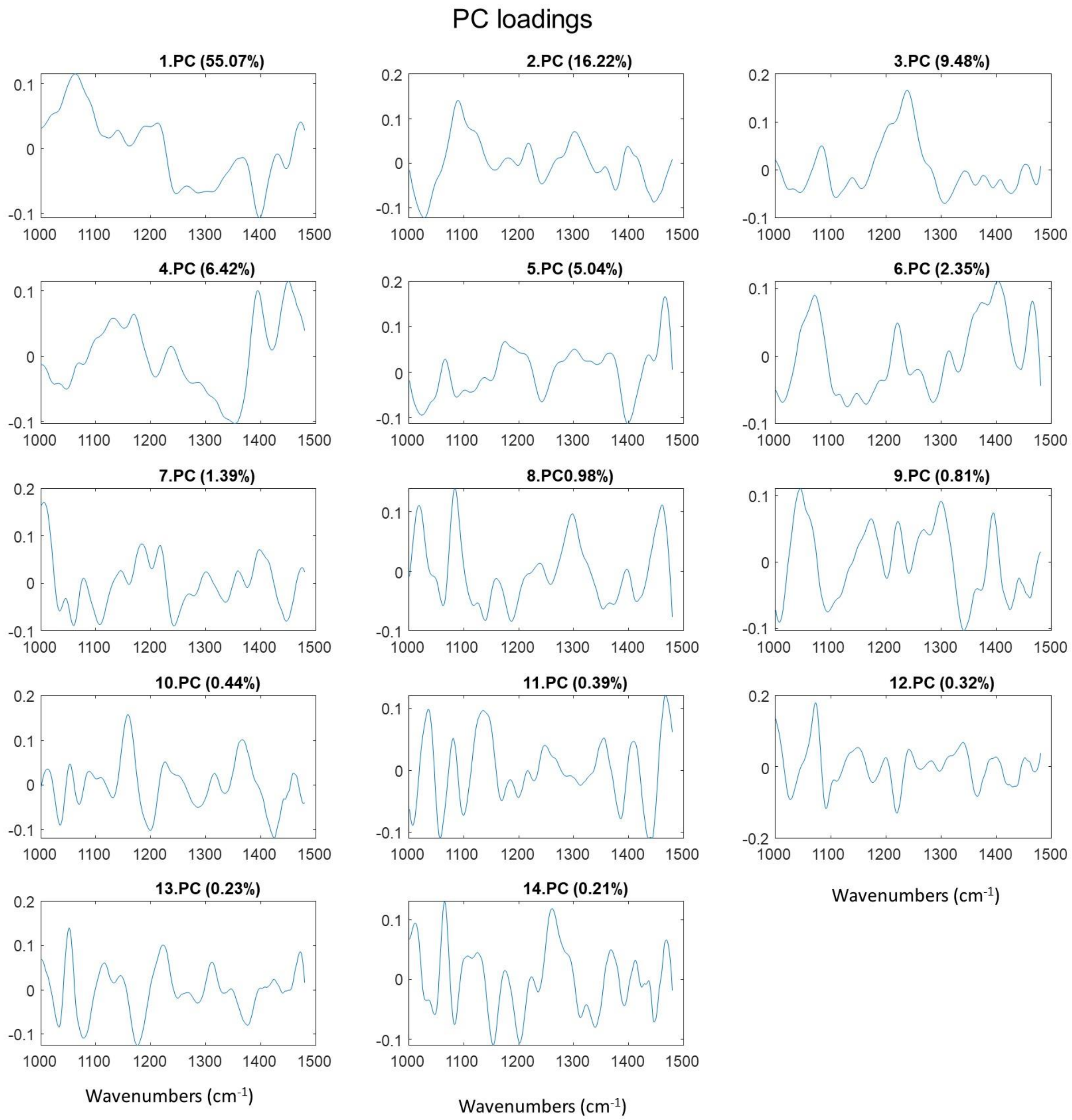

Figure S2: Principal component loadings of spectra of the training set. The percentage of the total variance explained by each principal component is stated

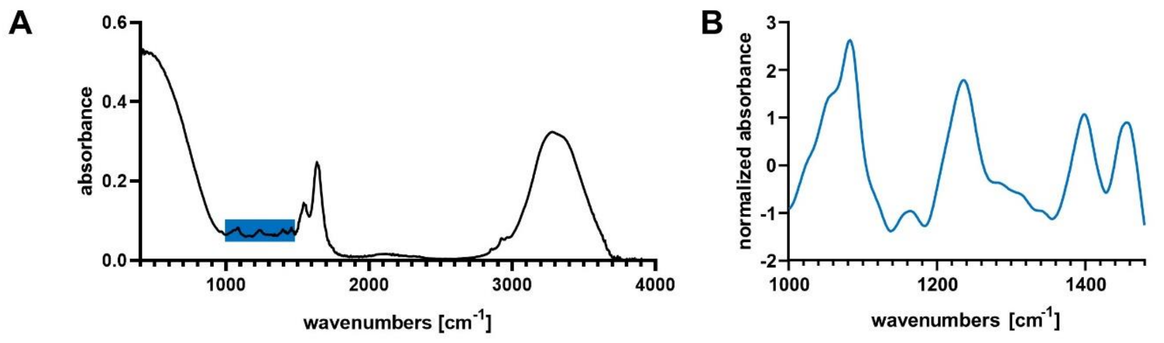

Figure S3: ATR FT-IR spectroscopy of fresh tissue. A: Raw spectrum as acquired **B:** After preprocessing. Only a small region of the original spectrum is used (blue box in A). The spectrum was obtained from a fresh biopsy of a metastasis of lung cancer (#270)

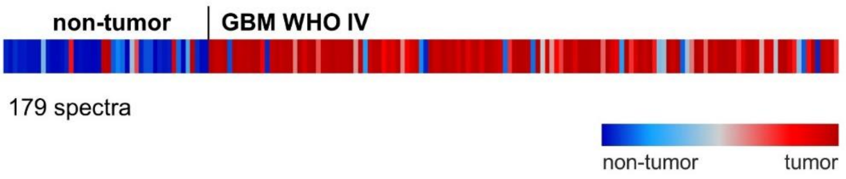

Figure S4: Reclassification of the spectra of the training set. The scores of the principal components 1-14 were used as input.

Analysis of misclassified specimens

Correct classified glioma WHO III spectra were characterized by a spectral pattern similar to that of GBM (Supporting Figure S5A, orange and red difference spectra). The spectra of glioma WHO III that were misclassified, on the other hand, lack the strong reduction of band at 1050 cm^{-1} and 1470 cm^{-1} and the increase at around 1240 cm^{-1} (Supporting Figure S5A, black spectra). Moreover, the data confirmed that the interval between tissue removal and IR spectroscopy did not affect the classification result (Supporting Figure S5B).

For glioma WHO III, clinical data was analyzed to identify possible disease-related causes for misclassification. However, no differences were found regarding 1p19q co-deletion and primary or recurrent disease (Supporting Figure S5B). Overall and progression-free survival were also similar (Supporting Figure S5C)

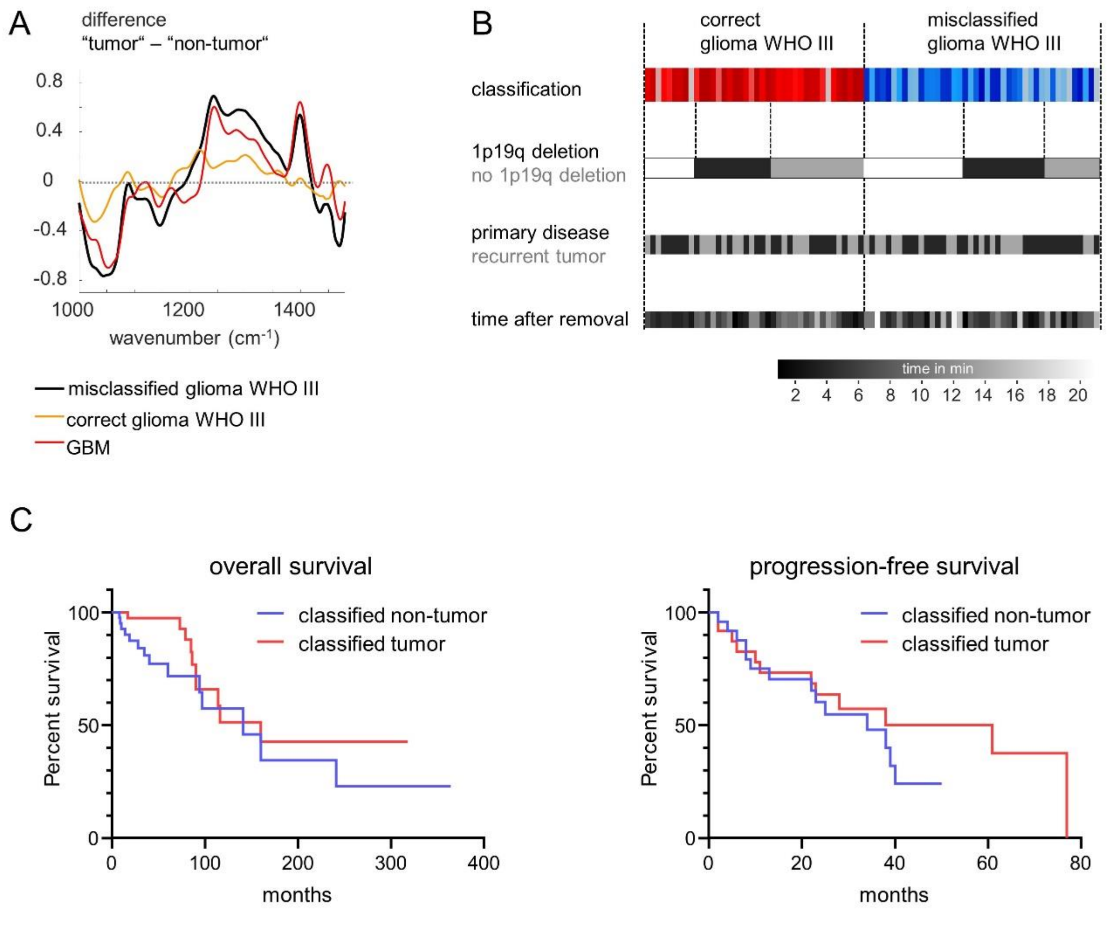

Figure S5: Analysis of misclassified specimens for glioma WHO III **A:** Spectral characteristics of correctly classified and misclassified specimens in comparison to GBM WHO IV. **B:** Comparison of the classification result of glioma WHO III to clinical data and experimental condition. **C:** Survival data is shown separately for the patients whose specimens were classified as "non-tumor" (=misclassified spectra) and whose specimens were classified as "tumor" (= correct classified spectra). Overall survival and progression-free survival were not different among groups (Log-rank (Mantel-Cox) test).

For GBM of the test set, the group of other primary brain tumors and brain metastases in total 55 samples of 511 were recognized as non-tumor, i. e. the result of the classification was not in accordance with the patient's diagnosis. Those specimens were analyzed in the same timeframe after resection (median five minutes 25% percentile: 3 min, 75% percentile: 10 min) compared to correctly classified samples. Therefore, other possible reasons were evaluated to better understand the potential and the limitations of our approach by analyzing the spectral signature of these samples (Supporting Figure S6). No spectral artifacts were found, confirming that these spectra were correctly acquired on the tissue sample. The comparison with the mean spectra of GBM and non-tumor tissue shows that misclassified spectra share in fact the same spectral characteristics with the non-tumor specimens. The difference to spectra of non-tumor samples is very small, while the pattern of the difference spectrum of GBM WHO IV and misclassified samples resembles the difference spectrum of GBM and non-tumor tissue (compare Figure 1). Histopathological analysis of the spectroscopically analyzed tissue (that was not identical with the one used for clinical diagnosis of the patient) revealed that 18 samples represented the tumor border and one sample reactive tissue, suggesting that the classification algorithm correctly identified regions of non-tumor tissue in those cases. The remaining 36 specimens were clearly rated as tumor by histopathology (Supporting Table T2). However, we cannot rule out the presence of non-tumor areas within the specimen, as no complete serial sectioning was performed.

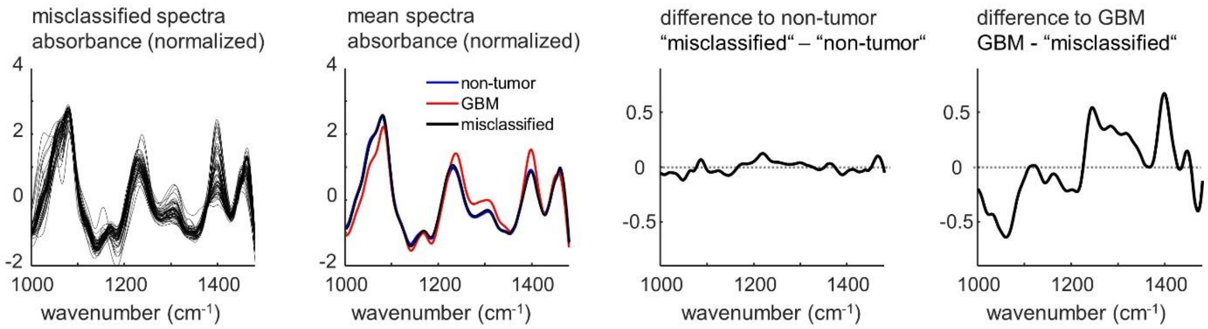

Figure S6: Analysis of misclassified specimens for brain tumors except glioma WHO II/III: Spectral characteristics of misclassified GBM, other tumors and brain metastases in comparison to spectra of non-tumor tissue and GBM.

Table S1. Assignment of bands of the infrared spectra to bond vibrations and biomolecules. According to [1]–[5]

Band position [cm^{-1}]	Vibration mode	Assingement
1030	ν(CO)	carbohydrates
1050	ν(COC), ν(COH)	carbohydrates, lipids
1080	$\nu_s(PO_2^-)$	phosphate groups; DNA, RNA
1200	$\nu_{as}(PO_2^-)$	phosphate groups
1240	amide III	proteins
1300	amide III	proteins
1400	$\delta_s(CH_3)$	proteins
1460	$\delta_{as}(CH_{2/3})$	lipids, proteins

[1] A. Blume, "Properties of lipid vesicles: FT-IR spectroscopy and fluorescence probe studies," *Curr. Opin. Colloid Interface Sci.*, vol. 1, no. 1, pp. 64–77, Feb. 1996, doi: 10.1016/S1359-0294(96)80046-X.

[2] I. Dreissig, S. Machill, R. Salzer, and C. Krafft, "Quantification of brain lipids by FTIR spectroscopy and partial least squares regression," *Spectrochim. Acta. A. Mol. Biomol. Spectrosc.*, vol. 71, no. 5, pp. 2069–2075, Jan. 2009, doi: 10.1016/j.saa.2008.08.008.

[3] R. K. Dukor, "Vibrational Spectroscopy in the Detection of Cancer," in *Handbook of Vibrational Spectroscopy*, American Cancer Society, 2006. doi: 10.1002/0470027320.s8107.

[4] C. Krafft and J. Popp, "Vibrational Spectroscopic Imaging of Soft Tissue," in *Infrared and Raman Spectroscopic Imaging*, John Wiley & Sons, Ltd, 2014, pp. 111–152. doi: 10.1002/9783527678136.ch3.

[5] Z. Movasaghi, S. Rehman, and D. I. ur Rehman, "Fourier Transform Infrared (FTIR) Spectroscopy of Biological Tissues," *Appl. Spectrosc. Rev.*, vol. 43, no. 2, pp. 134–179, Feb. 2008, doi: 10.1080/05704920701829043.

Table S2: Misclassified samples with spectral characteristics of non-tumor tissue. Diagnosis of the patient, histopathology for the specimen analyzed by IR spectroscopy, time after resection and probability of class assignment are given.

ID	diagnosis of patient	histopathology of measured sample	time after resection	Probability of class assignment
**55**	GBM WHO IV	tumor border	4	0.33
**119**	GBM WHO IV	tumor	2	0.47
**128**	GBM WHO IV	tumor border	5	0.18
**180**	GBM WHO IV	tumor	15	0.06
**206**	GBM WHO IV	tumor	9	0.23
**263**	GBM WHO IV	tumor border	17	0.18
**266**	GBM WHO IV	tumor border	19	0.36
**401**	GBM WHO IV	tumor	3	0.43
**457**	GBM WHO IV	tumor	12	0.06
**555**	GBM WHO IV	tumor border	3	0.00
**570**	GBM WHO IV	tumor border	6	0.19
**598**	GBM WHO IV	tumor	4	0.14
**602**	GBM WHO IV	tumor	19	0.02
**622**	GBM WHO IV	tumor border	2	0.08
**639**	GBM WHO IV	tumor	2	0.16
**719**	GBM WHO IV	tumor	5	0.32
**743**	GBM WHO IV	tumor		0.37
**780**	GBM WHO IV	tumor	2	0.13
**900**	GBM WHO IV	tumor		0.06
**15**	GBM WHO IV, recurrent	tumor border	6	0.44
**104**	GBM WHO IV, recurrent	tumor	24	0.46
**174**	GBM WHO IV, recurrent	tumor	10	0.43
**226**	GBM WHO IV, recurrent	tumor border	8	0.15
**377**	GBM WHO IV, recurrent	tumor border	1	0.36
**432**	GBM WHO IV, recurrent	tumor	5	0.05
**542**	GBM WHO IV, recurrent	tumor	6	0.14
**627**	GBM WHO IV, recurrent	tumor	6	0.40
**636**	GBM WHO IV, recurrent	reactive tissue	35	0.13
**650**	GBM WHO IV, recurrent	tumor	3	0.13
**769**	GBM WHO IV, recurrent	tumor	1	0.37
**828**	GBM WHO IV, recurrent	tumor	2	0.14
**934**	GBM WHO IV, recurrent	tumor border		0.21
**950**	GBM WHO IV, recurrent	tumor	16	0.23
**1156**	GBM WHO IV, recurrent	tumor	5	0.13
**1198**	GBM WHO IV, recurrent	tumor border	10	0.13
**1526**	GBM WHO IV, recurrent	tumor	9	0.32
**139**	Neuronal and mixed neuronal-glial tumours	tumor border	5	0.34
**711**	Neuronal and mixed neuronal-glial tumours	tumor	2	0.33

**896**	Neuronal and mixed neuronal-glial tumours	tumor border	3	0.17
**962**	Lymphomas	tumor	3	0.12
**1106**	Ependymal tumours	tumor	5	0.00
**1242**	Neuronal and mixed neuronal-glial tumours	tumor	1	0.15
**1493**	Neuronal and mixed neuronal-glial tumours	tumor	2	0.39
**185**	Metastatic tumours, lung	tumor	14	0.43
**600**	Metastatic tumours, lung	tumor	10	0.05
**847**	Metastatic tumours, lung	tumor border		0.45
**1023**	Metastatic tumours, lung	tumor border	3	0.23
**1074**	Metastatic tumours, lung	tumor	4	0.39
**1168**	Metastatic tumours, lung	tumor border	2	0.28
**1509**	Metastatic tumours, lung	tumor border	6	0.47
**842**	Metastatic tumours, melanoma	tumor	8	0.18
**1418**	Metastatic tumours, melanoma	tumor	10	0.48
**81**	Metastatic tumours, stomach	tumor	20	0.39
**725**	Metastatic tumours, laryns	tumor	7	0.02
**1214**	Metastatic tumours, unknown	tumor	3	0.41
